# Comparison of QT dispersion in patients with ST elevation acute myocardial infarction (STEMI) before and after treatment by streptokinase versus primary percutaneous coronary intervention (PCI)

**DOI:** 10.1186/s12872-020-01767-9

**Published:** 2020-11-23

**Authors:** Abbas Valizadeh, Sahar Soltanabadi, Saeed Koushafar, Maryam Rezaee, Reza Jahankhah

**Affiliations:** 1 Department of Cardiology, Fasa University of Medical Sciences, Fasa, Iran; 2 Student Research Committee, Fasa University of Medical Sciences, Fasa, Iran; 3 Dermatology Department, Molecular Dermatology Research Center, Shiraz University of Medical Sciences, Shiraz, Iran; 4 Department of Radiology, Medical Imaging Research Center, Shiraz University of Medical Sciences, Shiraz, Iran

**Keywords:** Myocardial infarction, Streptokinase, Primary PCI, QT dispersion

## Abstract

**Background:**

QT dispersion (QTD) represents inhomogeneous ventricular repolarization such that an increased QTD may predispose the heart to malignant ventricular arrhythmias (VAs). This study was conducted to compare QTD in patients with ST-elevation myocardial infarction (STEMI) before and after treatment by streptokinase (SK) versus primary percutaneous coronary intervention (PCI).

**Methods:**

The present case–control study was conducted on 185 STEMI patients who received SK (115 cases) or underwent primary PCI (70 cases). QTD and QT corrected dispersion before and 24 h after treatment. Likewise, they were also found to correct fatal arrhythmias (VT and VF) during the first 24 h after admission, and ejection fraction (EF) 24 h after treatment was evaluated.

**Results:**

QTD decreased in the primary PCI group, though no significant difference was seen between the two studied groups (*P* > 0.05). A significant increase was detected in the EF mean values for the primary PCI-treated patients (*P* = 0.022). Moreover, there was a significant reduction in QTD of patients with fatal arrhythmias in the primary PCI group (*P* = 0.022).

**Conclusion:**

An overall QTD reduction in the primary PCI group and a significant decrease in QTD of patients with fatal arrhythmias in the primary PCI group show that this treatment strategy is more efficient than thrombolytic therapy. As an important indicator of proper myocardial function, EF can independently predict improved myocardial function in the primary PCI group.

## Introduction

Acute myocardial infarction (AMI) is one of the most prevalent and life-threatening diseases worldwide. According to findings of various studies, an estimated annual number of 1.8 million people develop AMI in America. The probability of developing thrombosis in patients with ST-elevation myocardial infarction (STEMI) is more than 90%. Thus, reperfusion is the most important treatment for patients with STEMI [1, 2].

Primary percutaneous coronary intervention (PCI) and fibrinolysis (via fibrinolytic drugs such as streptokinase) are the two treatment choices with the aim of reestablishing blood flow to the ischemic tissues [3]. Performed within the first 1–2 h of the disease occurrence, PCI tries to provide a fast and effective blood supply to the ischemic vessels. In addition, provided that fibrinolytic therapy is used properly and in time, it will result in significantly reduced mortality and morbidity rates in patients [4].

Defined as the difference between the longest (QTmax) and the shortest (QTmin) QT intervals within a 12‐lead ECG, QT interval dispersion (QTD) is presently regarded as a diagnostic criterion for a cardiac autonomic tone which can be altered during ischemia. It is, moreover, an indicator of ventricular depolarization, accompanied by an increased risk of cardiac arrhythmia and sudden death in more severe stages. In some cases, in spite of numerous attempts for establishing reperfusion of the blocked vessels, ventricular repolarization power may not return to the primary stage due to the damage to the left ventricle caused by coronary artery occlusion [5, 6].

This study was conducted aiming to compare QTD in patients with STEMI before and after they were treated by streptokinase and those who were treated by primary PCI. Besides, this study also addressed the efficacy of the use of streptokinase and primary PCI in QT interval changes. Likewise, the use of QTD, as one of the cardiac arrhythmia-predicting methods in patients under treatment by streptokinase or primary PCI following STEMI, was also taken into consideration.

## Methods

### Study population

The present case–control study was conducted on 185 patients with STEMI hospitalized in Valiasr Hospital, Fasa, Iran. Briefly, out of 200 patients, consecutively admitted to the ED, 15 subjects were excluded on the account that they met the exclusion criteria. Thus, a total of 185 individuals were included, and then were divided into two groups (SK; n = 115 and PCI; n = 70). The criteria for inclusion in the study were as follows: patients’ visiting the emergency department (ED) of the hospital with retrosternal chest pain or discomfort which lasted for at least 30 min. Their condition was in such a way that two of their primary consecutive electrocardiograms (EKGs) showed an elevated ST segment; and also those whose lab data displayed increased levels of creatine kinase-MB (CK-MB) and troponin T. However, to prevent any deviation and inconsistency in the results of the study, patients with a 48-h symptom persistence period, EKGs showing SRQ duration of more than 120 s (right or left bundle branch block), any history of taking any kinds of medicine which affected SRQ duration or the heart rate, as well as a confirmed diagnosis of heart diseases such as congestive heart failure and/or valvular diseases that could produce changes to the QT interval dispersion or the heart rate, and moreover, those with cardiomyopathies and heart valve-related diseases were excluded from the study. The study was carried out in compliance with the edicts of the *Declaration of Helsinki* and obtained the approval of the Ethics Committee of Fasa University of Medical Sciences, Fasa, Iran (93132). All the participants signed the written informed consents form. Study by Cavusoglu et al. [7] was used as our base for references and considering QTD as our primary outcome. Given that the effect size is too large (ES = 1.26), and with respect to a power of 80% and Type-I error of 5%, the minimum sample size was estimated to be very low (11 participants in each group). We further decreased the effect size in our calculations to detect a minimum difference (ES = 0.5 as a medium effect size based on Cohen’s criteria) which resulted in minimum sample sizes of 128, 64 in each group. We used the following sample size calculation formula to determine the minimum sample size:

$$n = \frac{{2(Z_{1 - \alpha /2} + Z_{1 - \beta } )^{2} }}{{ES^{2} }}, \quad ES = \frac{MD}{\sigma }$$

### Determination of ST-elevation myocardial infarction

STEMI is defined as a new ST-segment elevation at the J point in contiguous leads with the cutoff point as greater than 0.1 mV, on 12-lead surface ECG. Location of the MI is determined through the area of the myocardium that is depicted by contiguous leads. For example, ST segment elevation in leads II, III, and aVF is defined as inferior MI. In addition to elevated ST, to diagnose STEMI, an increase in the circulating levels of cardiac enzymes is required. Troponin T level of at least one value above the 99th percentile upper reference limit; and/or a two-fold increase in the second measurement of CK-MB was done at 6 h apart from the first one [8], according to the patients’ records.

### Determination of QT interval and QT interval dispersion

In order to determine QT interval (QTc: QT corrected), 12-lead EKGs were obtained upon patients’ arrival to ED and 24 h after they received streptokinase or primary PCI. QT interval was calculated according to the formula presented below. In case a U wave was present, QT interval was calculated in accordance with the interval between U and T waves.

$$\hbox{QTc}=\frac{QT}{\sqrt{R-R interval}}$$

The QTD is the difference between the longest (QTmax) and the shortest (QTmin) QT intervals within a 12‐lead ECG. In the same order, QTc interval dispersion is the difference between the least and the most QTc intervals.

### Reperfusion therapy

In this study, the emergency cardiologist selected reperfusion therapy as the primary PCI or in the form of fibrinolytic drugs (pharmacotherapy) based on patients’ clinical status. Primary PCI was performed by a cardiac interventionist in the cath lab of Valiasr hospital, Fasa, Iran. At the time when symptoms emerged, pharmacological reperfusion was performed within 60 min or PCI within 90 min. It should be noted that the ideal symptom-to-needle time in SK group was within 30 min, but since some patients did not go to the ED on time, the maximum time was, at most, 60 min for some of our patients. Moreover, a cardiologist supervised and monitored the process of patients' receiving fibrinolytic drugs.

### Statistical methods of analyzing results

All the statistical analyses were performed using SPSS statistical software version 20.0 for Windows (SPSS Inc.). All the continuous variables were reported as mean ± standard deviation (M ± SD), and categorical variables were presented as number (percentages). The variables were compared between the two groups by means of student’s t-test/Mann–Whitney *U*-test and chi-square/Fisher’s exact test. The differences between the groups (more than two groups) were analyzed using one-way analysis of variance (ANOVA) with post-hoc Tukey as well as chi-square test. Paired t-test was also used to determine QTD mean differences occurring before and after therapeutics. P-value < 0.05 was considered statistically significant.

## Results

Based on the patients’ clinical status and decisions made by the cardiologists, 70 patients underwent primary PCI (58 men and 12 women, mean age = 62.50 ± 13.90 years) and 115 patients took fibrinolysis (76 men and 39 women, mean age = 62.10 ± 12.82 years).

There were no significant differences between the two studied groups in terms of age and sex (P = 0.40 and P = 0.62, respectively). Other variables such as cigarette smoking, hyperlipidemia, diabetes, history of CAD, and hypertension did not show significant differences between both therapeutic groups (P > 0.05) (Table 1).

**Table 1:** Comparison of basic and clinical characteristics between PCI and SK groups in STEMI patients

	Total (n = 185)	Primary PCI (n = 70)	SK (n = 115)	P-value*
Age		62.50 ± 13.90	62.10 ± 12.82	0.40
Sex				
Male	137 (74.1)	57 (81.4)	80 (69.6)	0.74
Female	48 (25.9)	13 (18.6)	35 (30.4)	
Hypertension				
Yes	71 (38.4)	25 (35.7)	46 (40.0)	0.56
No	114 (61.6)	45 (64.3)	69 (60.0)	
Diabetes				
Yes	31 (16.8)	8 (11.4)	23 (20.0)	0.13
No	154 (83.2)	62 (88.6)	92 (80.0)	
Hyperlipidemia				
Yes	50 (27.0)	24 (34.3)	26 (22.6)	0.08
No	135 (73.0)	46 (65.7)	89 (77.4)	
Smoking				
Yes	42 (22.7)	16 (22.9)	26 (22.6)	0.96
No	143 (77.3)	54 (77.1)	89 (77.4)	
History of CAD				
Yes	12 (6.48)	4 (5.71)	8 (6.95)	0.77
No	160 (93.52)	66 (94.29)	107 (93.05)	
Heart failure, (EF < 60%)				
Yes	27 (15.4)	7 (10.9)	20 (18.0)	0.21
No	148 (84.6)	57 (89.1)	91 (82.0)	

Continuous variables were reported as mean ± standard deviation (M ± SD), and categorical variables were presented as number (percentages)

*PCI* percutaneous coronary intervention, *SK* streptokinase, *STEM* ST-elevation myocardial infarction, *CAD* coronary artery disease, *EF* ejection fraction

Statistical significance where p < 0.05

^*^Comparison between the two groups

**Fig. 1:**
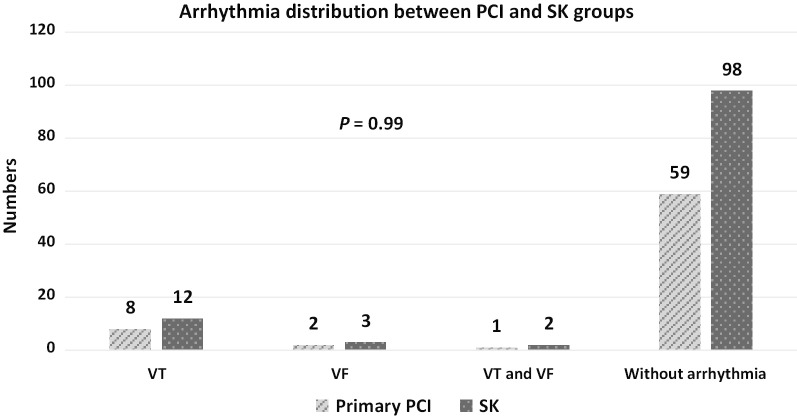
Arrhythmia distribution between percutaneous coronary intervention (PCI) and streptokinase (SK) therapies in ST-elevation myocardial infarction (STEMI) patients. Abbreviations: VT, ventricular tachycardia; VF, ventricular fibrillation. *P-value demonstrates the comparison among the four different categories

**Fig. 2:**
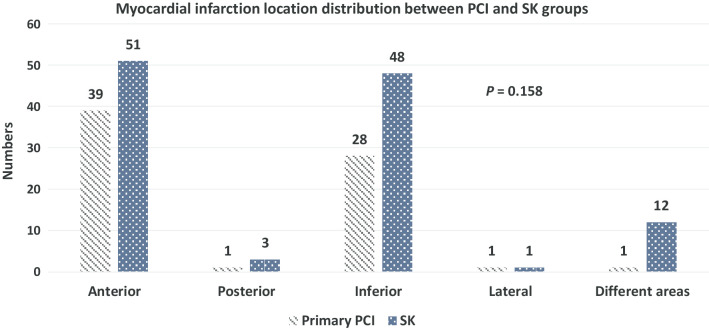
Myocardial infarction location distribution between percutaneous coronary intervention (PCI) and streptokinase (SK) therapies in ST-elevation myocardial infarction (STEMI) patients. *P-value demonstrates the comparison among the five different categories

In the present study, patients were categorized in terms of different types of arrhythmias as depicted in Fig. 1. A number of 28 patients were reported to have developed arrhythmias such that five had ventricular fibrillation (VF), 20 had ventricular tachycardia (VT), and three developed both VF and VT. There was no significant difference in the distribution of arrhythmia types between the two PCI and SK therapeutic groups (P = 0.99 for all comparisons). In addition, Fig. 2 illustrates the comparison of MI distribution between PCI and SK patients. The majority of patients (n = 90; 48.6%) experienced MI in their anterior area of the heart (39 for PCI and 51 for SK groups), and some in their inferior region (n = 76; 41%) (28 for PCI and 48 for SK groups). No significant difference was found between the two groups in terms of MI location distribution (P = 0.15 for all comparisons).

Table 2 depicts the comparison of QTD values among different arrhythmia types subsequent to both PCI and SK therapies. Patients who underwent PCI therapy (P = 0.02 for all comparisons), particularly those with VT and VF, showed a significant decrease in the mean value of their QTD. However, there were no significant differences in QTD after streptokinase therapy (P = 0.48 for all comparisons). It is worth mentioning that patients with arrhythmias showed QTD mean values of 64.28 ± 27.41 and 61.42 ± 33.52 before and after treatment, respectively (the data is not shown). The comparison of QTD values after both PCI and SK therapies among different MI locations is presented in Table 3. As it can be seen, no significant QTD changes were found in either PCI (P = 0.88) or SK (P = 0.81) method among different MI locations.

**Table 2:** Comparison of QTD mean values among different arrhythmia types after using PCI and SK therapies in STEMI patients

	Frequency	Mean	Standard deviation	95% CI		P-value*
PCI						
VT	8	10.00	35.45	− 19.64	39.64	0.022
VF	2	− 20.00	28.28	− 274.12	234.12	
VT and VF	1	− 120.00	–	–	–	
Total	11	− 7.27	50.01	− 40.87	26.32	
SK						
VT	12	− 6.66	33.39	− 14.55	27.88	0.489
VF	3	− 13.33	46.18	− 128.07	101.40	
VT and VF	2	− 20.00	28.28	− 274.12	234.12	
Total	17	0.00	34.64	− 17.81	17.81	

*QTD* QT dispersion, *PCI* percutaneous coronary intervention, *SK* streptokinase, *STEM* ST-elevation myocardial infarction, *VT* ventricular tachycardia, *VF* ventricular fibrillation, *95% CI* 95% confidence interval

Statistical significance where *P* < 0.05

*Comparison among the three different categories

**Table 3:** Comparison of QTD mean values among different MI locations after using PCI and SK therapies in STEMI patients

	Frequency	Mean	Standard deviation	95% CI		P-value*
PCI						
Anterior	39	2.05	47.63	− 13.39	17.49	0.887
Posterior	1	–	–	–	–	
Inferior	28	− 11.42	76.86	− 41.23	18.37	
Lateral	1	0.00	–	–	–	
Different sites	1	− 40.00	–	–	–	
Total	70	− 4.00	60.20	− 18.35	10.35	
SK						
Anterior	51	− 1.56	53.04	− 16.48	13.34	0.817
Posterior	3	26.66	46.18	− 88.07	141.78	
Inferior	48	1.66	45.16	− 11.44	14.78	
Lateral	1	0.00	–	–	–	
Different sites	12	− 10.00	34.64	− 32.00	12.00	
Total	115	− 0.34	47.53	− 9.12	8.43	

*QTD* QT dispersion, *MI* myocardial infarction,* PCI* percutaneous coronary intervention, *SK* streptokinase, *STEM* ST-elevation myocardial infarction, *EF* ejection fraction, *95% CI* 95% confidence interval

Statistical significance where *P* < 0.05

*Comparison among the five different categories

QTD values in PCI and SK groups were compared before and after treatment using paired t-test, the results of which are presented in Table 4. No significant difference was observed in the QTD mean values before (63.76 ± 51.93) and after (59.71 ± 28.79) PCI therapy (P = 0.58). Likewise, there was no significant difference in the QTD mean values before (60.86 ± 36.83) and after (60.52 ± 32.81) PCI therapy (P = 0.93). Moreover, using paired t-test analysis, no significant change was found in QTD mean values between the two studied groups before and after PCI and SK therapies (P = 0.93). Besides, no significant difference in QTc dispersion was seen between the two groups before and after PCI and SK therapies (P = 0.772). However, ejection fraction (EF) values showed a significantly higher mean level in patients treated by PCI (46.25 ± 9.08) in comparison with streptokinase ones (42.48 ± 11.11) (P = 0.02) (the data is not shown).

**Table 4:** Comparison of QTD mean values in STEMI patients before and after PCI and SK treatments through paired *t*-test

	Mean	Standard deviation	*t*	*P*-value*
PCI				
Before treatment	63.76	51.93	0.55	0.580
After treatment	59.71	28.79		
SK				
Before treatment	60.86	36.83	0.08	0.934
After treatment	60.52	32.81		
PCI	− 4.00	60.20	− 0.45	0.934
SK	− 0.34	47.53		

*QTD* QT dispersion,* PCI* percutaneous coronary intervention, *SK* streptokinase, *STEM* ST-elevation myocardial infarction, *EF* ejection fraction, *95% CI* 95% confidence interval

Statistical significance where *P* < 0.05

^*^Comparison between the two groups

## Discussion

The present study was performed to compare the QTD values in STEMI patients before and after they were treated by primary PCI and those were treated by SK. The findings revealed that there was no significant difference in QTD mean values of patients with STEMI before and after being treated by streptokinase compared to those under treatment by primary PCI. Furthermore, both PCI and SK therapies reduced QTD, though it is not statistically significant.

### QTD and QTc intervals values between primary PCI and thrombolytic groups

In our study, no significant decreases were seen in QTD and QTc interval values in primary PCI or thrombolytic groups. In line with the present study, the study performed by Oni Heris et al. showed that there was no significant difference in QTD before, one hour, and four days after treatment by streptokinase [13]. Also, no significant reduction was seen in patients treated by PCI. Furthermore, George et al. found that a decrease in QT and QTc interval dispersions after treatment by the thrombolytic method was not significant. In contrast, after treatment by primary PCI, a significant reduction was reported in QT and QTc interval dispersions compared to when the treatment was not commenced [14]. Moreover, the amount of decrease in QT and QTc interval dispersion was more significant in primary PCI than in thrombolytic therapy which was not in agreement with present findings. On the other hand, Mehta et al. presented results similar to those of our study. They found no significant reduction in QTD after treatment with thrombolytics [15]. However, Kobusiak-Prokopowicz et al. conducted a cohort study in which patients undergoing thrombolytic therapy were monitored for one year. Thus, they suggested a significant QTD reduction in those receiving thrombolytics [16]. In addition, Nikiforos et al. [17] evaluated the effects of thrombolytic or primary transluminal angioplasty treatments on QTD. Their thrombolysis and angioplasty groups showed a significant reduction in QT and QTc interval dispersions before and immediately after treatment which coincided with the results of the study conducted by Cavusoglu et al. [7]. Another study by Giedrimiene et al. showed the same findings, indicating that QTD was significantly reduced three days after successful primary PCI. Nevertheless, these results were not significant in unsuccessful primary PCI [18]. All in all, according to the findings of this study and the mentioned ones, it is now explicit that an appropriate myocardial reperfusion can be achieved via PCI therapy.

### Effects of primary PCI and thrombolytic therapies on arrhythmias occurrence

It is reported that in AMI patients, there is a connection between long QT syndrome (LQTS) and hypertrophic cardiomyopathy prolonged QTD, on the one hand, and a higher risk of malignant ventricular arrhythmias (VAs), on the other [19]. In fact, an increase in QTD will bring about a decrease in the integrity of ventricular repolarization. It is shown that the effective treatment and management of MI and arrhythmias can lead to decreased QT [9, 20]. In this research, QTD mean values in patients with arrhythmias were reduced before and after treatment. On the other hand, QTD changes in patients with arrhythmia showed a significant reduction after PCI therapy in comparison with the fibrinolysis group, suggesting that the primary PCI method is superior to thrombolytic in treating MI and reducing its life-threatening complications such as dangerous ventricular arrhythmias (i.e.; VT, VF). Findings obtained from Kobusiak-Prokopowicz et al. did not show any significant differences in the incidence of tachycardia with fibrinolytic treatment after one year [16]. However, the findings obtained by Van de Loo et al. demonstrated significantly greater QTD levels in those with VF during the first 24 h than those without arrhythmias [21].

### Association of QT interval dispersion and MI location in primary PCI and thrombolytic groups

The present study addressed the relationship between the changes in QT interval dispersion and MI location. The results did not show a significant decrease in QT interval dispersion changes in relation to MI locations. The study by George et al. revealed that changes in QT and QTc interval dispersion in terms of infarct location (anterior or inferior MI) were not significant as well [14]. On the other hand, one of the results obtained in the study conducted by Cavusoglu et al. [22] and Hassan [23] indicated that according to the patients' initial ECGs at admission time, the values of QT and QTc dispersion in patients with anterior MI were higher than those with inferior MI. Moreover, contrary to our findings, Cavusoglu observed a significant reduction in QT and QTc interval dispersions in both infarction locations after reperfusion therapy. It is noteworthy that QT and QTc dispersions are believed to rely on the size of infarct. Likewise, it is revealed that a larger infarction could account for the high QT and QTc dispersion values which are related to anterior MI [23].

### Effects of primary PCI and thrombolytic therapies on ejection fraction

An important outcome of our study can be said to be the investigation of cardiac function. In addition, we found a significant difference between EF mean in the two therapeutic groups, such that primary PCI group showed a significantly higher value compared to the fibrinolysis group. Thus, it can consequently represent the more effective and higher quality of treatment with primary PCI method. Also, an evaluation of the relationship between EF (as an index of cardiac function) and reduced QTD was a unique point of view in our study.

### Limitations

This study had its own limitations. Given that QT values were computed manually, it gives rise to the possibility of biased measurement. Given the probable risk of miscalculations being performed by only one person; we not only assigned two people to compute every value separately, but we also turned the data over to an expert to triple check. Thus, to obtain more robust and more precise measures, a computer should be used, if available. Second, medications that are likely to undermine the calculation of QT values could not be standardized at the time of patients' enrollment. The third was the absence of any course of long-term follow-up for VA development in these patients. It would be also of an enormous use to compare the acute QTD changes with other arrhythmogenic markers (late potentials, heart rate variability) and other electrocardiographic markers of successful reperfusion, such as a prompt decrease in ST elevation. It is recommended that further studies be also conducted on the QTD value of an ECG calculated after angiography in the fibrinolysis group. Yet, one of the advantages of the present study is the large sample size, mostly ignored by other similar researches. Moreover, comparing the effects of the two therapeutic methods, i.e. primary PCI and pharmacotherapy, on QTD changes is another strong point of our study.

## Conclusion

In the present study, no significant difference was seen between primary PCI and thrombolytic therapy in decreasing QT and QTc interval dispersions after treatment. However, it was found that QTD values in primary PCI method experienced a greater reduction. As increased QT interval can pave the way for the development of dangerous arrhythmia, as this study showed a significantly reduced QTD in patients with arrhythmia treated by primary PCI and also significantly recovered cardiac function in PCI method; all could suggest the priority of this method over the thrombolytic therapy.

## Data Availability

The dataset analyzed during the current study are available from the corresponding author on reasonable request.

## Abbreviations

AMI: Acute myocardial infarction
ANOVA: One-way analysis of variance
CAD: Coronary artery disease
CK-MB: Creatine kinase-MB
ED: Emergency department
EF: Ejection fraction
EKG: Electrocardiograms
MI: Myocardial infarction
PCI: Percutaneous coronary intervention
QTc: QT corrected
QTD: QT dispersion
SK: Streptokinase
SD: Standard deviation
STEMI: ST elevation acute myocardial infarction
VA: Ventricular arrhythmia
VF: Ventricular fibrillation
VT: Ventricular tachycardia
